# Effect of *HbDHN1* and *HbDHN2* Genes on Abiotic Stress Responses in Arabidopsis

**DOI:** 10.3389/fpls.2017.00470

**Published:** 2017-04-10

**Authors:** Yuxin Cao, Xian Xiang, Mengting Geng, Qin You, Xi Huang

**Affiliations:** Hainan Key Laboratory for Sustainable Utilization of Tropical Bioresources, Institute of Tropical Agriculture and Forestry, Hainan UniversityHaikou, China

**Keywords:** *Hevea brasiliensis*, dehydrin, abiotic stress, overexpression, laticifer differentiation

## Abstract

Dehydrin is a type of late embryogenesis abundant (LEA) protein. The dehydrin genes, *HbDHN1* and *HbDHN2*, in *Hevea brasiliensis* were previously found to be induced at the wounding site of epicormic shoots, with local tissue dehydration identified as the key signal for laticifer differentiation. However, the exact role of the HbDHNs remains unknown. In this study, *HbDHN1* and *HbDHN2* expression was examined under multiple abiotic stresses; namely, cold, salt, drought, wounding, abscisic acid (ABA), ethylene (ET), and jasmonic acid (JA) treatment. Although, both HbDHNs were defined as SK2-type dehydrin, they showed different cellular localizations. Overexpression of the HbDHNs in *Arabidopsis thaliana* further revealed a significant increase in tolerance to salt, drought and osmotic stresses. Increased accumulation of proline and a reduction in electrolyte leakage were also observed under salt and drought stress, and a higher water content was indicated under osmotic stress. The transgenic plants also showed higher activity levels of ascorbate peroxidase (APX), superoxide dismutase (SOD) and catalase, and accumulated less hydrogen peroxide (H_2_O_2_) and superoxide (${\text{O}}_{2}^{-}$). Given that reactive oxygen species (ROS) are thought to be a key signal for laticifer differentiation, these findings suggest that HbDHNs act as ROS scavengers, directly or indirectly affecting laticifer differentiation. Both HbDHNs therefore influence physiological processes, improving plant tolerance to multiple abiotic stresses.

## Introduction

Plants are subjected to various abiotic stresses from low temperature and drought to salinity. To survive, they have evolved several different mechanisms to enhance their overall tolerance, including stress signal perception and transduction, and associated molecular regulatory networks. Products of the genes involved in these mechanisms are classified into two groups according to their function during stress (Hirayama and Shinozaki, 2010). The first group contains proteins involved in the regulation of signal transduction and stress-inducible gene expression, and includes transcription factors and protein kinases (e.g., MAP kinases, phosphatases, and phospholipid metabolic enzymes). The second includes proteins that function in direct abiotic stress tolerance such as chaperones, late embryogenesis abundant (LEA) proteins, osmotin, and detoxification enzymes (Shinozaki et al., 2003).

Dehydrins (DHNs) belong to the group 2 LEA protein family. They were redefined based on the existence of at least one copy of a highly conserved 15-amino acid lysine-rich K-segment (EKKGIMDKIKEKLPG) forming an amphiphilic a-helix (Koag et al., 2009; Hanin et al., 2011). However, conservation of the K-segment is not absolute and even the most conserved residues in its core (Lys-Ile-Lys-Glu) are sometimes substituted (Graether and Boddington, 2014). Accordingly, DHN proteins are divided into five subclasses: Kn, KnS, YnKn, SKn, and YnSKn, based on the number of conserved K-, S-, and Y-segment motifs (Close, 1996). The Y-segment (DEYGNP) shares high homology with the nucleotide binding site motif of chaperone proteins and is located near the N-terminus of the DHN protein, while the S-segment includes a block of serines with a protein phosphatase binding site at the end (Graether and Boddington, 2014). Recently, 54 DHN genes were identified in the wheat expressed sequence tags database, encoding seven types of dehydrin (KS, SK3, YSK2, Y2SK2, Kn, Y2SK3, and YSK3). Of these, YSK2 and Kn were shown to be more represented in the genome of wheat than any other cereal (Wang et al., 2014).

Dehydrins function as stress proteins during protective reactions against dehydrative stresses in a number of plant species such as wheat and barley (Brini et al., 2007a, 2010; Kosova et al., 2014a,b), *Physcomitrella patens* (Saavedra et al., 2006), *Arabidopsis* (Kovacs et al., 2008), maize (Hanin et al., 2011), *Dactylis glomerata* (Volaire, 2002; Volaire et al., 2005), *Trifolium repens* (Vaseva and Feller, 2013; Vaseva et al., 2014), and *Medicago truncatula* (Xie et al., 2012). Cryoprotective activity has also been widely reported; for example, WCS120 in common wheat (Houde et al., 1995), COR85 in spinach (Kazuoka and Oeda, 1994), and PCA60 in peach (Wisniewski et al., 1999). Furthermore, wheat dehydrin DHN-5 was found to improve the activity and thermos-stability of fungal β -glucosidase and glucose oxidase enzymes *in vitro*, suggesting that DHNs are also involved in heat tolerance (Brini et al., 2010). Moreover, DHNs help maintain cell volume and prevent cellular collapse due to their unfolded state, higher accumulation and ability to bind water under drought stress. In addition, the S-fragment of several DHNs contains a phosphorylation site; for example, a differential phosphorylation pattern was observed in DHN-5 during wheat response to drought and salt stress (Brini et al., 2007b). Improved responses to abiotic stress and resulting plant adaptation are therefore largely the result of DHNs, which help protect cells from dehydration, stabilize the cell membrane, eliminate free radicals, bind metal ions, and act as a molecular chaperone (Sun and Lin, 2010; Graether and Boddington, 2014).

*Hevea brasiliensis* is a major commercial source of natural rubber. Rubber latex is synthesized and accumulated in laticifer, the network structure of which develops from anastomosed laticifers and is arranged in rings parallel to the vascular cambium. Latex is expelled from a large area of bark through a single tapping cut (Hao and Wu, 2000). In young stems of epicormic shoots, there are only a few rings of primary laticifer; however, secondary laticifer differentiation can be induced from vascular cambia via mechanical wounding or treatment with exogenous jasmonic acid (JA) (Hao and Wu, 2000; Tian et al., 2003; Yu et al., 2007). The exploited trees produce two-to three-fold more laticifer rings than unexploited trees (Hao and Wu, 1982, 1984), suggesting that JA-mediated wounding responses are involved in laticifer differentiation and latex biosynthesis. A recent report further suggests that the differentiation of secondary laticifers is prevented when the wounding site of epicormic shoots is wrapped immediately after wounding. Wounding-induced laticifer differentiation therefore seems to be correlated with reactive oxygen species (ROS) and JA accumulation as well as dehydration (Tian et al., 2015).

We previously identified DHN genes *HbDHN1* and *HbDHN2* in *H. brasiliensis*, and revealed differentiated expression in wrapped and unwrapped wounding sites of epicormic shoots, confirming that dehydration at the wounding site is closely related to the wounding response (Cao et al., 2017); however, the details of this response remain unknown. For example, how HbDHNs affect the wounding response and laticifer differentiation and whether they have a specific role in the wounding response or in response to other abiotic stresses, such as cold, dehydration, salinity, and drought, is yet to be determined. In this study, GFP-fusion of *HbDHN1* and *HbDHN2* was therefore performed via Agrobacterium-mediated transformation of leaves and PEG-mediated transformation of protoplasts of *Nicotiana tobaccum*. Quantitative real-time PCR (qRT-PCR) was then performed to determine expression patterns in the latex, leaves, stem tip, bark, staminate flowers and pistillate flowers of *H. brasiliensis*. HbDHN transformations of Arabidopsis plants were also carried out, and the transgenic plants subjected to different conditions of abiotic stress were analyzed. Also, activity levels of ascorbate peroxidase (APX), superoxide dismutase (SOD) and catalase, and accumulation of hydrogen peroxide (H_2_O_2_) and superoxide (${\text{O}}_{2}^{-}$), were evaluated.

## Materials and methods

### Plant materials and treatments

*H. brasiliensis* Reran7-33-97 was grown in the experimental farm of the University of Hainan, Hainan, China. Pistillate and staminate flower, leaf, bark, latex, and stem tip samples were collected from adult rubber trees and immediately frozen in liquid nitrogen. Total RNA was isolated using the Plant RNA Isolation kit (BioTeke, China). Reran7-33-97 seedlings subjected to cold, salt, drought, wounding, H_2_O_2_, ABA, PEG, NaCl, ET, and MeJA treatments were cultivated in a climate chamber under the following conditions: 16 h light (100 lux)/8 h dark, 75% relative humidity, and a temperature of 27°C. Seedlings were then sprayed with 100 μmol l^−1^ MeJA, 1.5% ethrel, 2% H_2_O_2_, or 100 mmol l^−1^ ABA. For PEG and salt treatments, the seedlings were irrigated once with 20% PEG6000 or 200 mmol l^−1^ NaCl, and for cold treatment were transferred from 27 to 4°C. For wounding treatment leaves were scraped with forceps. Leaf samples were collected at 0, 3, 6, 12, 24, and 48 h post-treatments, respectively, snap frozen in liquid nitrogen then stored at −80°C until RNA extraction.

### Subcellular localization analysis

The full-length open reading frames (ORFs) of *HbDHNs* were fused in frame with GFP into the pCAMBA1300-GFP vector. The constructs were then transfected into Agrobacterium strain LBA4404 and infiltrated into leaves of *N*. *tobaccum* via Agrobacterium-mediated transformation. Alternatively, plasmids were transferred into the protoplast of *N. tobaccum* via PEG-mediated transformation as described previously (Liu et al., 2013). GFP fluorescence was visualized and photographed with a Laser Scanning Confocal Microscope (Olympus, FluoView FV1000).

### Transformation and treatment in arabidopsis

The full-length ORFs of *HbDHN1* and *HbDHN2* were amplified and inserted into the pCAMBIA1302 plasmid between *Nco*I and *Bst*EII, respectively. Agrobacterium-mediated transformation was performed according to the floral dipping technique of *A. thaliana* (ecotype Columbia). Transgenic plants were selected on MS agar plates supplemented with 50 mg/L hygromycin and homozygous T_3_ plants used for further treatments. *A. thaliana* seeds were surface sterilized for 5 min in 5% sodium hypochlorite, washed with distilled water three times, placed at 4°C for 2–4 d in the dark then planted on 1/2 MS agar medium (Sigma-Aldrich) supplemented with 1% agar and 1% sucrose, pH 5.8, at 22°C and illuminated under 16 h light/8 h dark conditions. After 5 days of cultivation, seedlings were transferred to vertical 1/2 MS plates supplemented with NaCl (100 and 150 mM, respectively) or mannitol (200 and 300 mM, respectively). Root length was measured 7 days after transplantation. For drought stress, 3-week-old seedlings were subjected to drought treatment by withholding water until a lethal effect of dehydration was observed. Three days after rewatering, surviving plants were counted and photographed.

### Quantitative real-time PCR (qRT-PCR)

Total RNA of Arabidopsis was isolated using TRIzol reagent (Invitrogen, Carlsbad, CA) and RNA of *H. brasiliensis* samples isolated using a Plant RNA Isolation kit (BioTeke, China). First-strand cDNA was synthesized using the RevertAid™ First-Strand cDNA Synthesis Kit (Fermentas, Lithuania). *Actin2* and *HbYLS8* or *Hb18S* (Li et al., 2011) were used as standard controls for Arabidopsis and *H. brasiliensis*, respectively. All primers used are listed in Table S1. Real-time RT-PCR was performed using the fluorescent dye SYBR Green (Takara, China) and analyzed with the 7,500 Real-Time PCR system (Applied Biosystems Industries, USA). Expression levels of the target gene was plotted as RQ (RQ = 2^−ΔΔCT^; Livak and Schmittgen, 2001). qRT-PCR conditions were set as follows: 30 s at 95°C for denaturation followed by 45 cycles of 5 s at 94°C and 30 s at 60°C for amplification. Three independent biological replicates were performed per treatment.

### Measurement of physiological indices

Rosette leaves were collected after each treatment and the relative water content (RWC) determined as described previously (Brini et al., 2007a). For measurements of water loss, 2-week-old seedlings were removed from the 1/2 MS growing medium and plated on dry filter paper. Water loss was then determined as the percentage of initial fresh weight. Free proline content, SOD and catalase activity were determined using Proline, SOD, and Catalase Assay Kits, according to the manufacturer's instruction, respectively (Nanjing Jiancheng Bioengineering Institute, China); APX activity was assayed as previously described (Mittova et al., 2000). *In situ* detection of H_2_O_2_ and ${\text{O}}_{2}^{-}$ determined using vacuum-infiltrating with 10^−2^ M nitro-blue tetrazolium (NBT) or 1 mg/mL diaminobenzidine (DAB) solution for 12 h and cleared in 75% ethanol, respectively, as previously described (Able, 2003).

## Results

### Bioinformatic analysis of the *HbDHN1* and *HbDHN2*

Full-length ORFs of *HbDHN1* and *HbDHN2* encoded 225 and 210 amino acid residues, respectively. Both contained an S segment (SSSSSSS) and two K segments (EKKGLKEKIKEKLPG). A phylogenetic tree grouped the HbDHNs with SK2-type DHNs (Figure S1), and included AAN78125, ABS12342.1, BAA04569.1, and ABD95986.1, which reportedly improve drought and cold tolerance (Yang et al., 2012). To further analyze gene regulation *in silico*, cis-acting elements in the HbDHN promoters were scanned using the online tool PlantCARE (plant cis-acting regulatory elements, http://bioinformatics.psb.ugent.be/webtools/plantcare/html/; Lescot et al., 2002). Several stress response-related cis-acting elements, including ABRE, DRE, TGACG palindrome, and LTR motifs, were revealed in both the *HbDHN1* and *HbDHN2* promoters (Table S2). These elements are involved in ABA, drought, low-temperature, and JA signaling pathways, suggesting that expression of *HbDHN1* and *HbDHN2* is regulated by multiple abiotic stresses.

### Subcellular localization of HbDHN1 and HbDHN2

**Figure 1:**
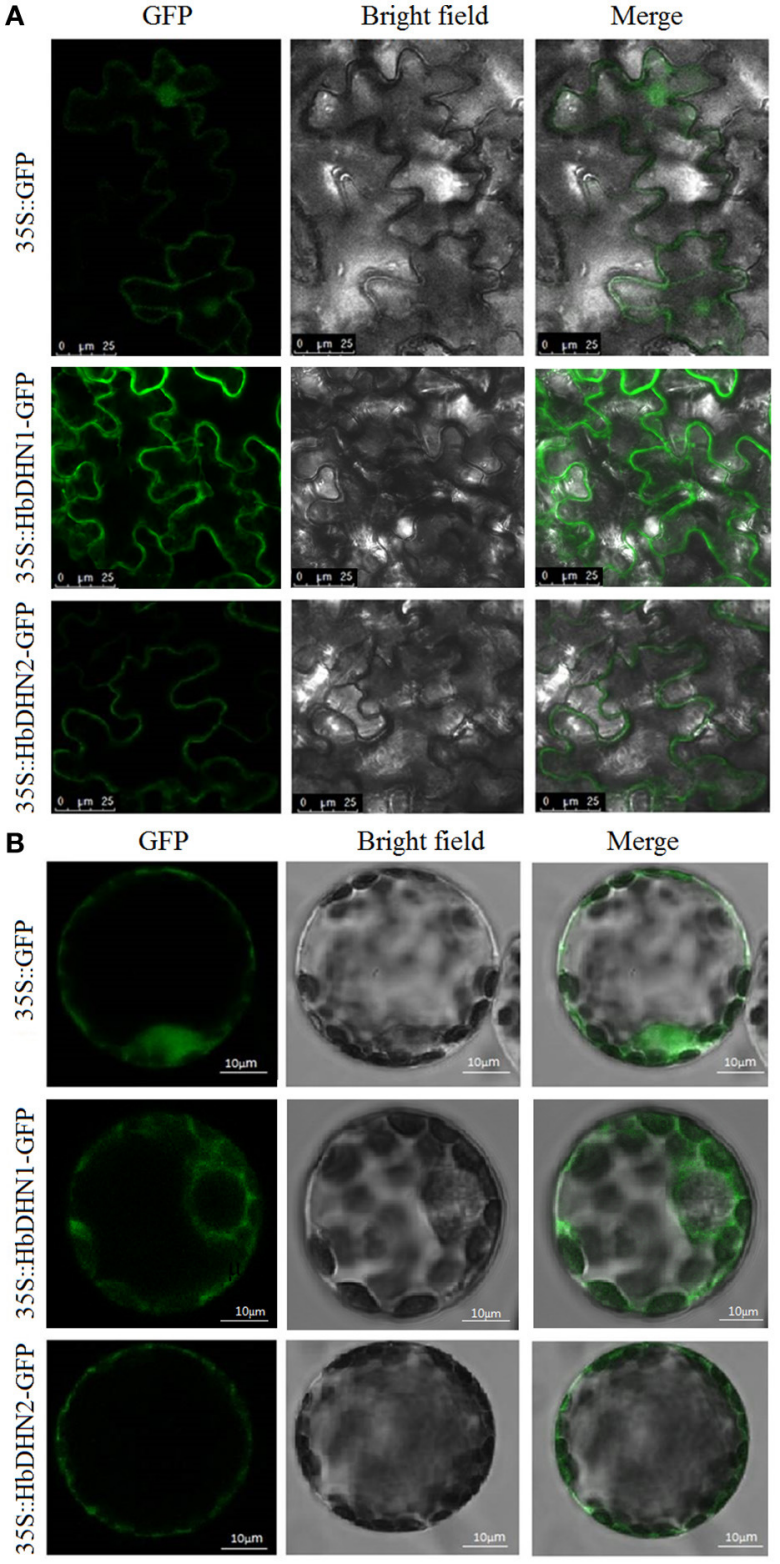
**Subcellular localization of *HbDHN1* and *HbDHN2***. Epidermis **(A)** and protoplasts **(B)** of transgenic tobacco plants expressing *35S:HbDHN1-GFP* and *35S:HbDHN2-GFP* fusion proteins observed under a laser scanning confocal microscope.

Subcellular localization of HbDHN1 and HbDHN2 was first determined via Agrobacterium-mediated transformation. Agrobacterium containing an HbDHN-GFP fusion construct was infiltrated into epidermis leaves of *N. tobaccum* and visualized by confocal fluorescence microscopy. Fluorescence of both the HbDHN1 and HbDHN2 fusion proteins was clearly localized at the edge of the cell, most likely the plasma membrane (Figure 1A). However, when constructs were transferred into the protoplast via PEG-mediated transformation, the HbDHN1-GFP fusion protein was clearly visible in the cytoplasm. However, the HbDHN2-GFP fusion protein remained at the edge of the cell and seemed to localize in the plasma membrane (Figure 1B). These findings suggest that the protoplast is more advantageous than the epidermis for GFP fusion protein localization and confirm that HbDHN1 and HbDHN2 are both SK2-type dehydrins; however, differentiated cellular localization suggests that they play different roles in cell physiological processes.

### Expression profiles of *HbDHN1* and *HbDHN2*

**Figure 2:**
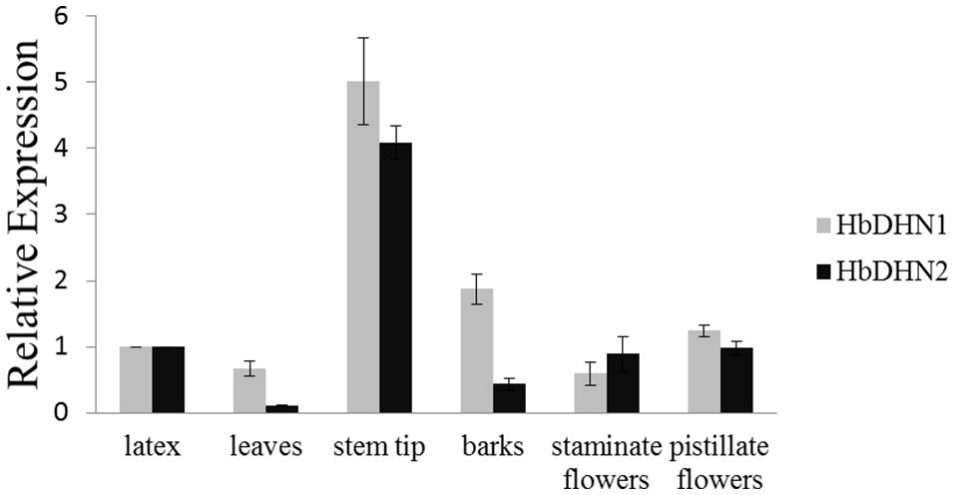
**Expression profiles of *HbDHN1* and *HbDHN2* in different tissues**. Error bars indicate the SE based on three replicates.

**Figure 3:**
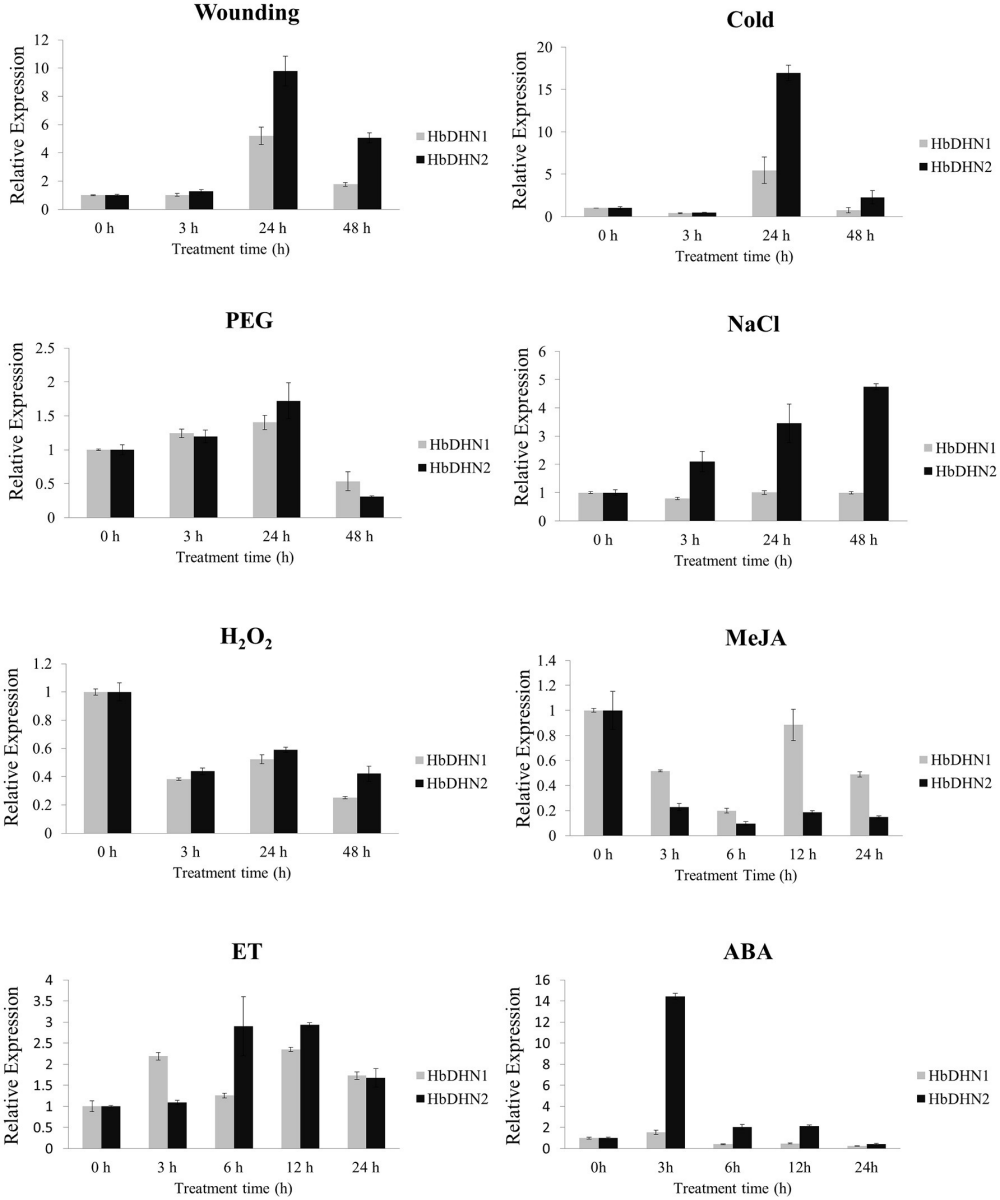
**Expression profiles of *HbDHN1* and *HbDHN2* after different stress treatments**. Seedlings were subjected to salt (200 mmol l^−1^ NaCl), cold (4°C), 20% PEG6000, wounding, H_2_O_2_ (2%), MeJA (100 μmol l^−1^), ET (1.5%), and ABA treatment (100 mmol l^−1^ ABA). Relative expression levels of *HbDHN1* and *HbDHN2* were detected by qRT-PCR at the indicated times. Error bars indicate the SE based on three replicates.

To determine tissue-specific expression patterns of *HbDHN1* and *HbDHN2*, qRT-PCR was used to detect expression in the latex, leaves, stem tip, bark, staminate flowers, and pistillate flowers, respectively. Both *HbDHN1* and *HbDHN2* showed highest transcript abundance in the stem tip, while HbDHN1 was also strongly expressed in the bark (Figure 2). To further investigate expression under different stress conditions, qRT-PCR of expression patterns under different treatments was performed. As shown in Figure 3, both *HbDHN1* and *HbDHN2* were significantly induced under wounding, cold, PEG, and ethylene treatment, respectively, and downregulated under H_2_O_2_ and JA treatment. *HbDHN2* was intensively induced by NaCl and ABA treatment, whereas expression of *HbDHN1* was less affected (Figure 3). These data suggest that although the expression patterns of *HbDHN1* and *HbDHN2* were similar under multiple abiotic stresses, their roles do not completely overlap.

### Overexpression of *HbDHN1* and *HbDHN2* enhances tolerance to osmotic stress

**Figure 4:**
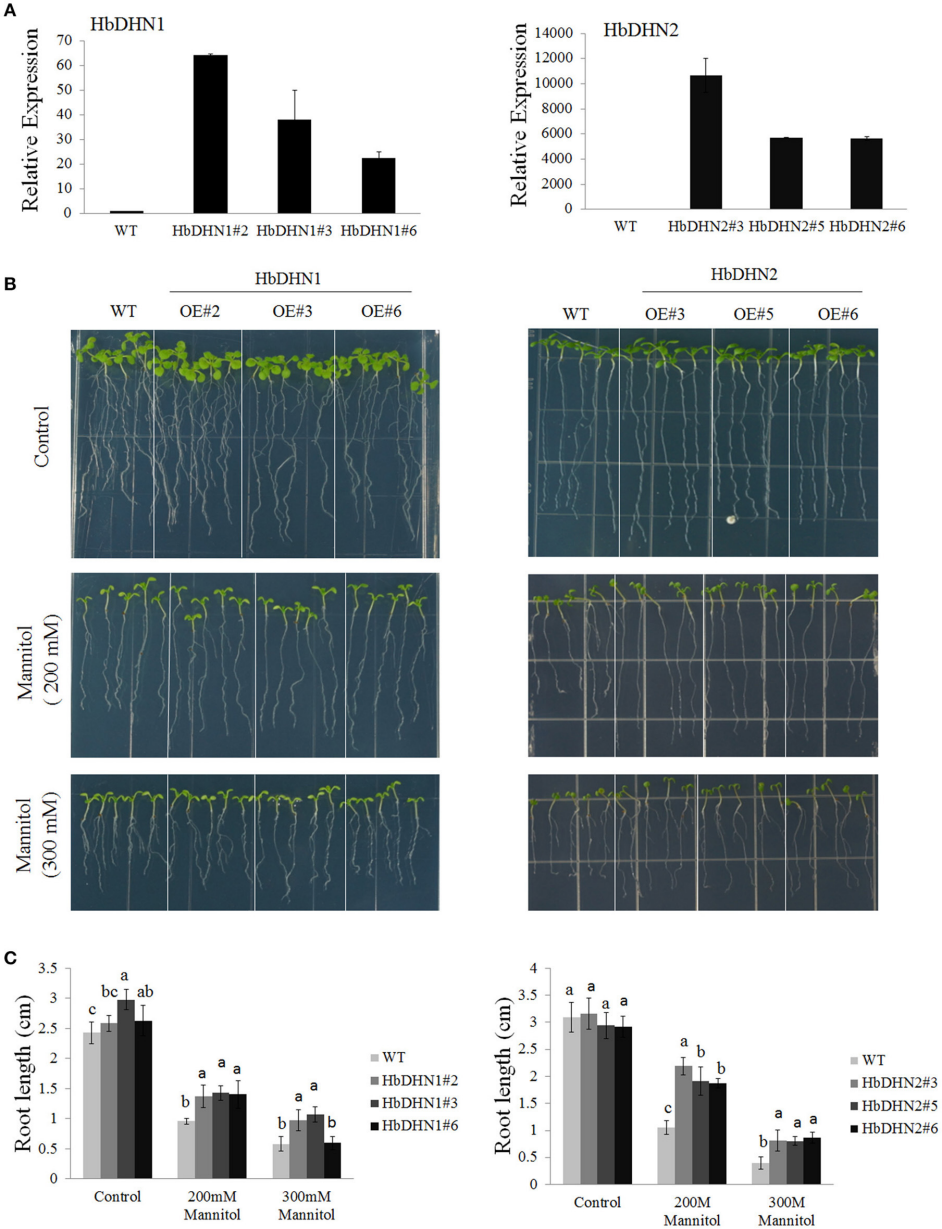
**Overexpression of *HbDHN1* and *HbDHN*2 enhances osmotic tolerance in Arabidopsis. (A)** Expression levels of *HbDHN*-overexpressing *A. thaliana*. **(B)** The seedlings were transferred onto 1/2 MS medium containing 200 or 300 mM mannitol. Phenotypes were observed; **(C)** The root lengths were measured at 7 days after transferring. Data represent mean values ± SE obtained from three independent experiments. Different letters indicate a significant difference at the level of *p* < 0.05. Four columns in a block (WT and three transgenic lines) were analyzed for significant differences.

To further determine their function, *HbDHN1* and *HbDHN2* were transformed and over-expressed in Arabidopsis. Three homozygous T_3_ lines were selected for each HbDHN and qRT-PCR performed. The results showed that all lines of both *HbDHN1* (OE#2, OE#3, and OE#6) and *HbDHN2* (OE#3, OE#5, and OE#6) were highly expressed in the transgenic Arabidopsis (Figure 4A).

To test osmotic tolerance, 5-day-old seedlings were transferred to 1/2 MS agar medium containing mannitol, further cultivated for 7 days and then root lengths were measured. When grown on medium without mannitol, no significant differences in root length were observed between the wild-type (WT) and transgenic plants. In contrast, root lengths of both sets of transgenic plants were significantly longer than that of the (wild-type) WT plants when grown on medium containing 200 or 300 mM mannitol, suggesting that the overexpression of *HbDHN1* and *HbDHN2* enhances osmotic tolerance (Figures 4B,C).

### Overexpression of *HbDHN1* and *HbDHN2* enhances drought tolerance

**Figure 5:**
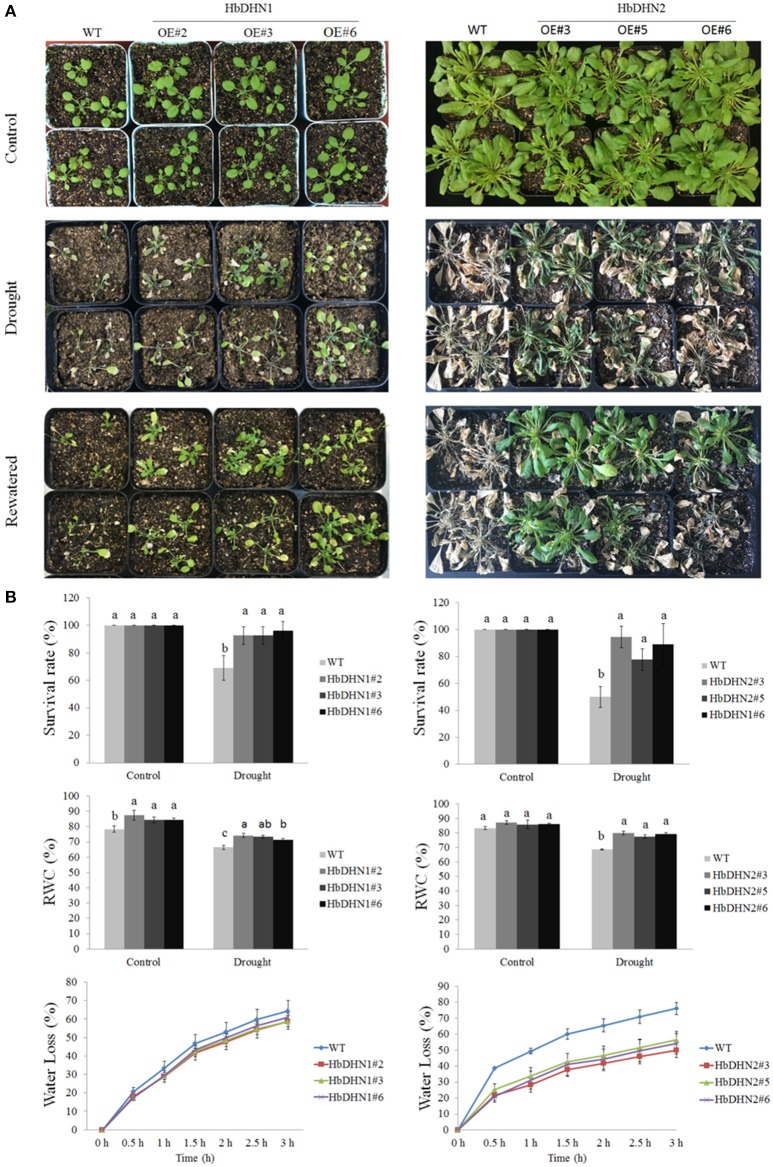
**Overexpression of *HbDHN1* and *HbDHN*2 enhanced drought tolerance in *A. thaliana*. (A)** Three-week-old seedlings were subjected to drought treatment by withholding water. Phenotypes were observed at 20 days after drought treatment and further 3 days after re-watering; **(B)** The survival rates were calculated. The relative water contents were measured at 7 days after treatment. The seedlings were removed from 1/2 MS plates to dry filter paper and water loss was measured at the indicated time points. Data represent mean values ± SE obtained from three independent experiments. Different letters indicate a significant difference at 0.05 level. Four columns in a block (WT and three transgenic lines) were analyzed for significant differences.

To investigate drought tolerance, 1-month-old seedlings underwent drought treatment for 15 successive days. Both transgenic and WT plants showed leaf wilting symptoms after drought treatment; however, symptoms were more serious in the WT (Figure 5A). Furthermore, survival rates of the *HbDHN*-overexpressing plants were significantly higher than the WT after 15 days of drought stress (Figure 5B). In addition, no obvious differences in the RWC of the leaves were observed between transgenic and WT plants (ca. 78–87% vs. 82–87%, respectively) under normal water conditions. However, at 10 days after drought treatment, leaf RWCs of both the *HbDHN1* and *HbDHN2* transgenic lines were significantly higher the WT (~71–74% and 77–79% vs. 66–68%, respectively; Figure 5B). This finding was further confirmed by water loss data: leaf water loss in the transgenic lines was significantly lower than the WT plants under drought stress (Figure 5B). When rewatered, recovery of the transgenic lines was much better than that of the WT (Figure 5A). Combined, these data suggest that overexpression of the *HbDHNs* increases water retention and enhances survival ability under drought stress.

**Figure 6:**
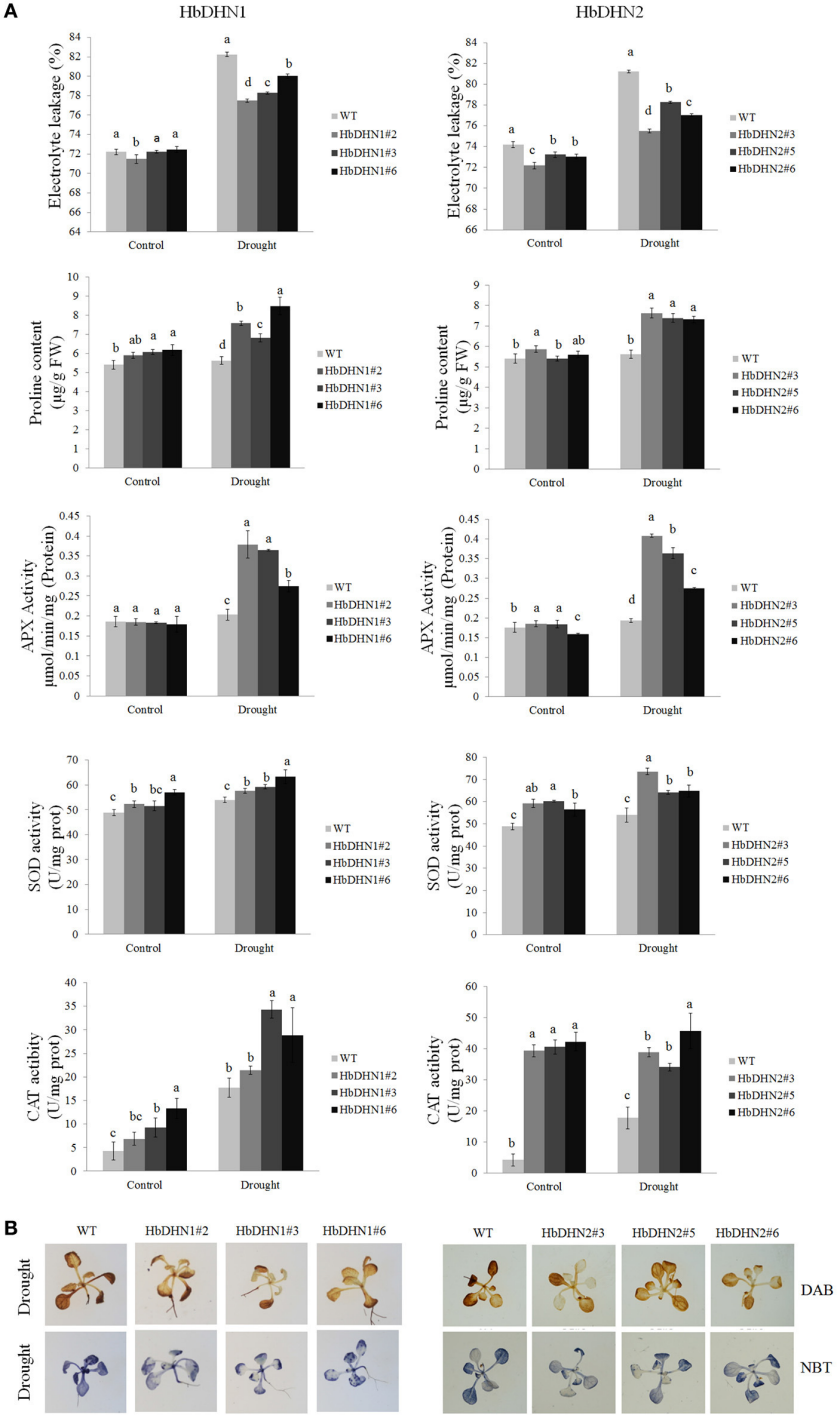
**Effect of drought stress on growth of wild-type (WT) and HbDHN transgenic *A. thaliana* seedlings. (A)** Three-week-old seedlings were irrigated with 20% PEG 6000; relative electrolyte leakage, proline content, APX activity, SOD activity and catalase (CAT) activity in leaves were determined 12 h after treatment. Data represent means ± SE of three biological replicate samples. Different letters indicate a significant difference at the level of *p* < 0.05. Four columns in a block (WT and three transgenic lines) were analyzed for significant differences. **(B)** Two-week-old seedlings irrigated with 20% PEG 6000 for 3 h; H_2_O_2_ and ${\text{O}}_{2}^{-}$ accumulation were indicated by DAB and NBT staining, respectively. Levels of blue and brown staining on leaves indicate the generation of ${\text{O}}_{2}^{-}$ and H_2_O_2_, respectively.

Furthermore, relative electrolyte leakage was higher in the WT than the *HbDHN1* and *HbDHN2* transgenic lines. The activity of antioxidant enzymes, such as APX, SOD, and catalase, increased more significantly in the *HbDHN1* and *HbDHN2* transgenic lines than in WT. The proline content also increased under drought stress, but the levels were also higher in the *HbDHN1* and *HbDHN2* transgenic lines than in the WT (Figure 6A). Accumulation of H_2_O_2_ and ${\text{O}}_{2}^{-}$ was also determined by DAB and NBT staining, respectively. As shown in Figure 6B, both the transgenic lines and WT displayed increased staining, indicating ROS accumulation under drought stress. However, compared to the WT, accumulation of both H_2_O_2_ and ${\text{O}}_{2}^{-}$ was lower in the *HbDHN1* and *HbDHN2* transgenic lines. These results suggest that overexpression of the *HbDHN* enhances plant drought tolerance by increasing the activity of antioxidant enzymes and proline content, leading to a decrease in ROS accumulation and cell electrolyte leakage.

### Overexpression of *HbDHN1* and *HbDHN2* enhances salt tolerance

**Figure 7:**
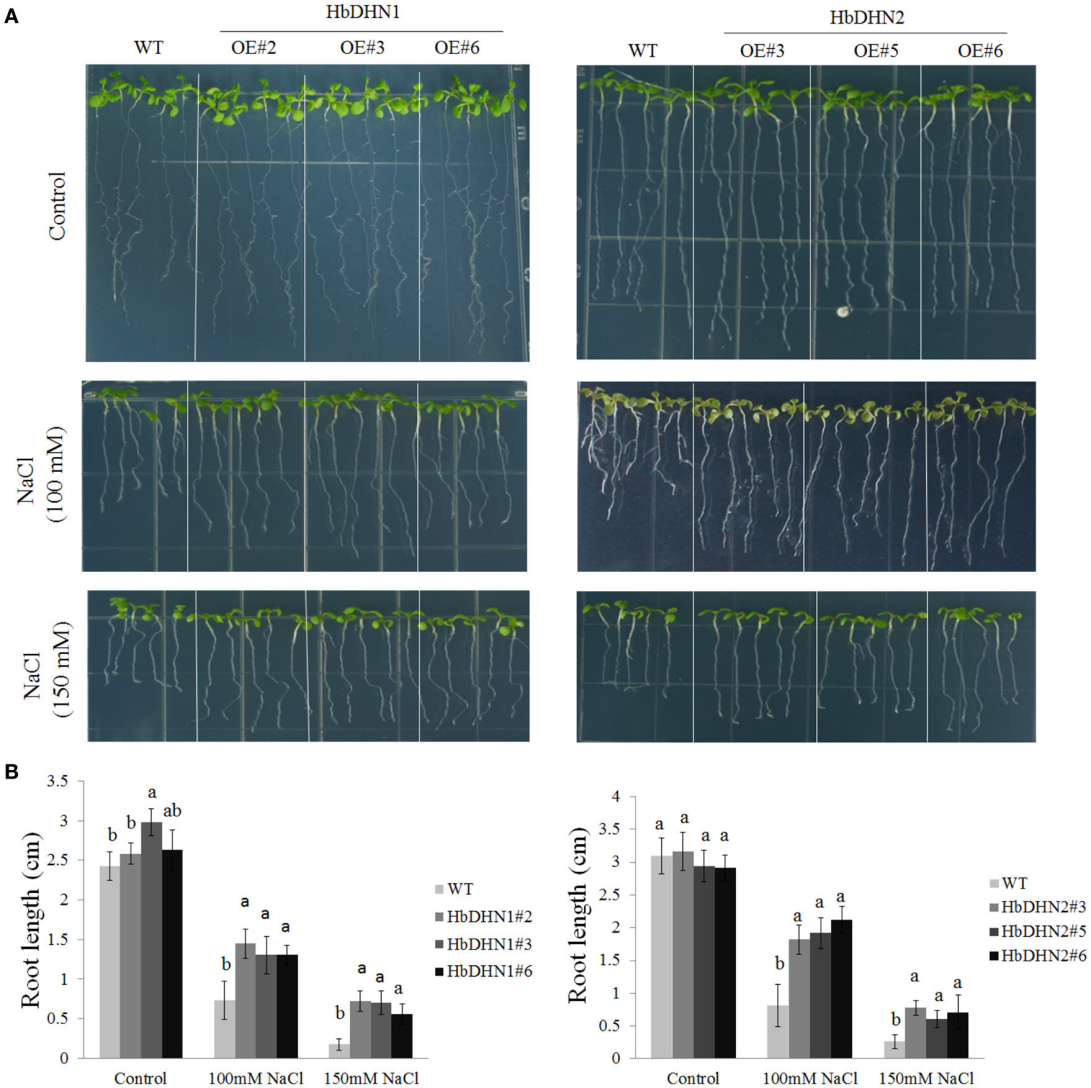
**Overexpression of *HbDHN1* and *HbDHN2* enhances salt tolerance in *Arabidopsis seedlings*. (A)** Wild–type (WT) and *HbDHN*-overexpressing *A. thaliana* were transferred onto 1/2 MS medium containing 100 or 150 mM NaCl, phenotypes were observed; **(B)** and root lengths were measured at 7 days after treatment. Data represent mean values ± SE obtained from three independent experiments. Different letters indicate a significant difference at the level of *p* < 0.05. Four columns in a block (WT and three transgenic lines) were analyzed for significant differences.

To test salt tolerance, 5-day-old seedlings of WT and T_3_ homozygous *HbDHN1* and *HbDHN2* lines were transferred to 1/2 MS agar medium with different concentrations of NaCl then root length measured at 7 days after treatment. There were no significant differences in root length between the WT and transgenic lines under normal growth conditions; however, under salt stress, root lengths of both transgenic lines were significantly longer than the WT (Figures 7A,B).

**Figure 8:**
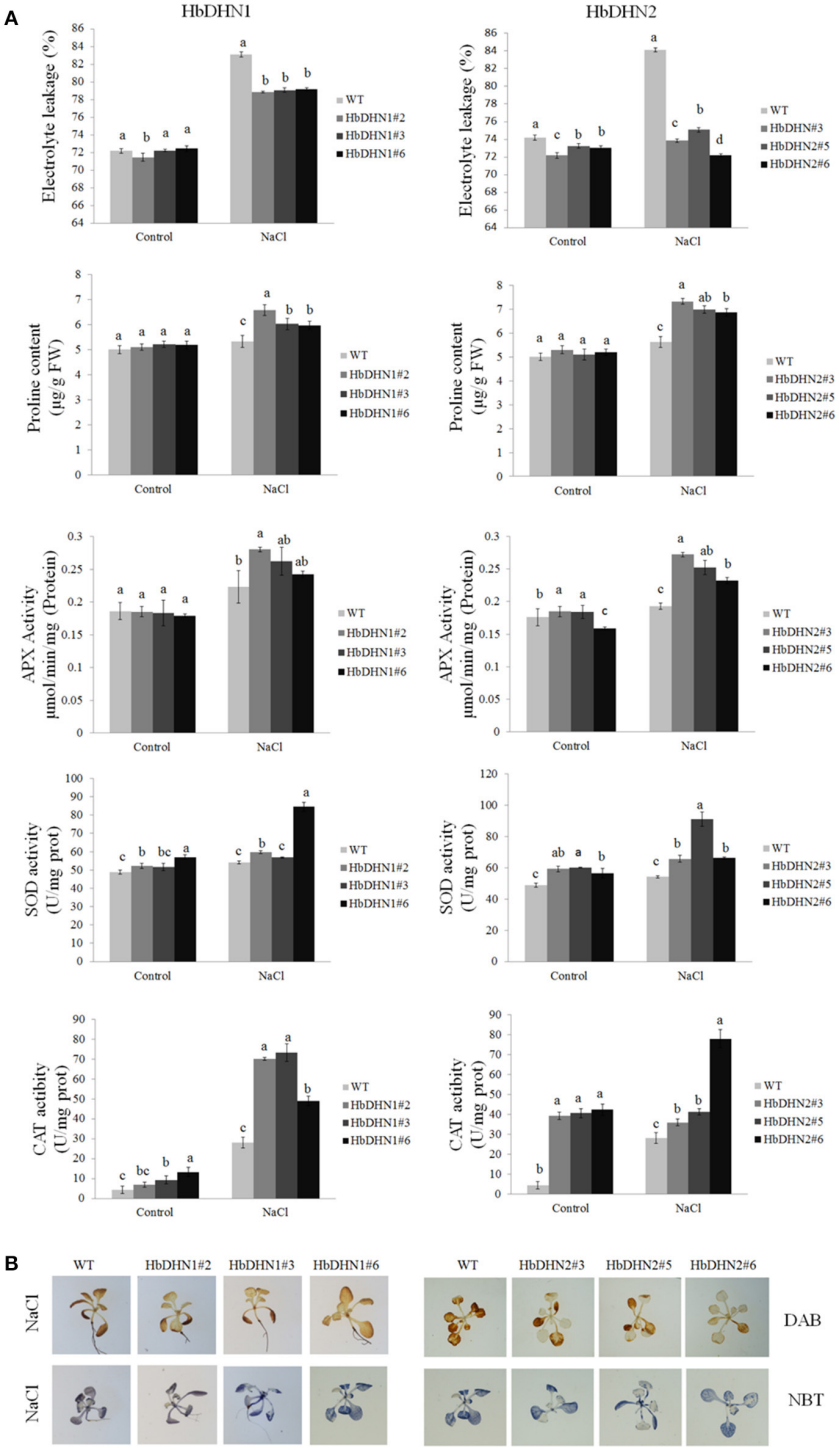
**Effect of salt stress on the growth of wild-type (WT) and HbDHN transgenic *A. thaliana* seedlings. (A)** Three-week-old seedlings were irrigated with 200 mM NaCl; relative electrolyte leakage, proline content, APX activity, SOD activity and catalase (CAT) activity in leaves were determined 12 h after treatment. Data represent means ± SE of three biological replicate samples. Different letters indicate a significant difference at the level of *p* < 0.05. Four columns in a block (WT and three transgenic lines) were analyzed for significant differences. **(B)** Two-week-old seedlings irrigated with 200 mM NaCl for 3 h; H_2_O_2_ and ${\text{O}}_{2}^{-}$ accumulation were indicated by DAB and NBT staining, respectively. Levels of blue and brown staining on leaves indicate the generation of ${\text{O}}_{2}^{-}$ and H_2_O_2_, respectively.

Similar to drought stress, the transgenic lines also showed a decrease in electrolyte leakage, higher activity levels of APX, SOD, and catalase, and a higher proline content than the WT under salt stress (Figure 8A). In addition, the WT accumulated more H_2_O_2_ and ${\text{O}}_{2}^{-}$ than both transgenic lines (Figure 8B). These results suggest that increased antioxidant enzymes activity and proline biosynthesis in the *HbDHN* transgenic lines caused a decrease in ROS accumulation and cell leakage, improving salt tolerance.

## Discussion

Mechanical wounding and treatment with exogenous JA were both found to induce secondary laticifer differentiation in young stems of epicormic shoots of rubber tree (Hao and Wu, 2000; Tian et al., 2003; Yu et al., 2007). A key step in revealing the underlying mechanisms of this response was the observation that laticifer differentiation is prevented when the wounding site is wrapped immediately after wounding (Tian et al., 2015). Two SK2-type dehydrins (HbDHN1 and HbDHN2) were previously found to be induced by dehydration of the wounding site, suggesting that mechanical wounding acts as a dehydration stress whereby cellular damage leads to water loss. In line with this, local tissue dehydration was proven to be a key signal of wounding-induced laticifer differentiation (Cao et al., 2017). In *Boea crassifolia*, Y*n*SK2-type dehydrin BcDh2 is reportedly induced by wounding, and moreover, wound-induced expression was found to be mediated by JA and salicylic acid (SA) signaling pathways (Shen et al., 2004). Furthermore, Y2K-type dehydrin CpDHN1 from *Cicer pinnatifidum* and S8K4-type dehydrin PgDHN1 from *Picea glauca* were also found to be induced by JA and methyl-JA (Richard et al., 2000; Bhattarai and Fettig, 2005), while cold-inducible Kn-type dehydrin Lti30 in *A. thaliana* (Xero 2) is also induced on wounding (Rouse et al., 1996). Distinct SKY motifs of wounding-induced dehydrins suggest that these DHNs play diverse roles in the wounding response. However, the specific roles of *HbDHN1* and *2* in the wounding response and laticifer differentiation remain unknown, and whether they act as specific dehydrins or function widely across different abiotic stresses requires further investigation.

Analysis of cis-acting elements in promoter regions showed that both the HbDHN1 and HbDHN2 promoters contain common cis elements such as ABRE (Kizis and Pages, 2002), ARE, DRE (Hao et al., 2002), TGACG and CGTCA palindrome motifs (Kim et al., 1993, 1994; Table S2). These findings suggest that expression is regulated by ABA-dependent and -independent signaling pathways. ABA-dependent signaling pathways include bZIP transcription activators known as AREBs (ABRE binding factors), which bind to ABRE elements; CBF4/DREB1D transcription activator, which binds to CRT/DRE/LTRE elements; and MYBFs and MYCFs, which bind to MYB and MYC promoter elements, respectively. ABA-independent signaling pathways include DREB2A and DREB2B transcription activators, which bind to CRT/DRE/LTRE elements (Zhu, 2002; Shinozaki et al., 2003; Yamaguchi-Shinozaki and Shinozaki, 2005). Previously, YnSKm-type DHNs in common wheat and Kn-type dehydrins DHN-5 in barley were found to be induced by both cold and drought as well as dehydration (salt and frost) and ABA due to the occurrence of several ABRE elements in their promoters (Choi et al., 1999; Tommasini et al., 2008; Wang et al., 2014). Furthermore, TGACG and CGTCA palindrome motifs were found to be essential for JA induction of promoters of the nopaline synthase (nos) gene in *Agrobacterium tumefaciens* T-DNA (Kim et al., 1993, 1994) and the barley lipoxygenase 1 gene (LOX1) (Rouster et al., 1997), suggesting that both HbDHNs are regulated by JA. These suggestions were further supported by the qRT-PCR results, whereby both *HbDHN1* and *HbDHN2* were significantly upregulated by ABA, wounding, cold, PEG and ethylene treatment, but downregulated by JA and H_2_O_2_ treatment. This confirms that the HbDHNs are not specific to the wounding response, but function widely during various abiotic stresses such as cold, dehydration, salinity, and drought (Figure 3). This is consistent with the previous suggestion that many SKn proteins are upregulated by cold, desiccation, and salt (Graether and Boddington, 2014).

The protein architecture of dehydrins can be described by the presence of three types of conserved sequence motif, K-, Y-, and S-segments. The presence or absence of the YSK motif is thought to be correlated with localization of the dehydrin in the cell as well as the particular abiotic stress that triggers its expression (Graether and Boddington, 2014). YnKn, YSK, and Kn dehydrins have been identified in the cytoplasm and the nuclei (Houde et al., 1995; Wisniewski et al., 1999; Lin et al., 2012), while SKn dehydrins are found near the plasma membrane (Danyluk et al., 1998; Hara et al., 2003) and one KnS dehydrin was found in the mitochondrial fraction (Hara et al., 2003). No dehydrin possessing the Y-segment has yet to be observed in the membrane, suggesting that the Y-segment does not play a role in membrane protection (Graether and Boddington, 2014). Both *HbDHN1* and *HbDHN2* contain one S fragment at the N-terminal and two K fragments at the C-terminal, and are therefore classified as SK_2_-type dehydrin (Cao et al., 2017). Here, both HbDHN1 and HbDHN2 were found near the plasma membrane following epidermal transformation (Figure 1A). However, when the fusion constructs were transferred into the protoplast via PEG-mediated transformation, the HbDHN1-GFP fusion protein was clearly visualized in the cytoplasm and the HbDHN2-GFP fusion protein appeared to localize in the plasma membrane (Figure 1B). Given that the thickness of a cell membrane is 3–10 nm, whether HbDHN2 located in the plasma membrane or probably in parietal cytoplasm requires further investigation, such as using *in situ* immunolocalization with transmission electron microscopy. In addition to the conserved YSK motifs, all residues located between the conserved Y-, S-, and K-segments are defined as Φ-segments. Since neither the sequences nor the lengths are conserved, the Φ-segment is a catchall definition (Graether and Boddington, 2014). Dehydrins with His–His or His flanking the K-segments reportedly have an effect on membrane binding (Eriksson et al., 2011). The results of this study suggest that the Φ-segments of the HbDHNs might affect the cellular localization.

To further understand how HbDHN1 and HbDHN2 affect plant responses to abiotic stresses, we generated transgenic Arabidopsis lines and analyzed their functions under abiotic stress. Overexpression of the *HbDHNs* improved salt and drought tolerance in the transgenic plants, increased water retention and enhanced survival under drought and osmotic stress (Figures 4, 5). The transgenic lines also had longer roots and lower electrolyte leakage, higher SOD, APX, and catalase activity, and higher proline content than the WT under drought and salt stress (Figures 6–8). In addition, the WT plants accumulated more H_2_O_2_ and ${\text{O}}_{2}^{-}$ than the *HbDHNs* transgenic lines, suggesting that overexpression increases antioxidant activity and proline biosynthesis, decreasing ROS accumulation and cell leakage, and thereby enhancing stress tolerance.

Tian et al. (2015) proposed that ROS is a key signal of laticifer differentiation in rubber trees, having observed inhibition of mechanical wounding-induced laticifer differentiation after treatment with diphenyleneiodonium chloride (DPI), a specific inhibitor of NADPH oxidase. In this study, since HbDHN overexpression in Arabidopsis enhanced antioxidant activity and decreased the accumulation of H_2_O_2_ and ${\text{O}}_{2}^{-}$ under drought and salt stress it is possible that the HbDHNs also act as a ROS scavenger, directly or indirectly regulating the redox status *in vivo* that affects laticifer differentiation.

Many DHNs containing relatively large amounts of His, Arg, and other reactive amino acid residues on their surface exhibit ROS scavenging properties. The citrus dehydrin CuCOR19 prevented the oxidation of liposomes, most likely by scavenging ROS (Hara et al., 2003). Such functions are mediated by direct interactions between the amino acid residues and ROS species (such as ${\text{O}}_{2}^{-}$, HO^−^, and H_2_O_2_), or via interactions with metal ions that lead to the formation of covalent bonds (Hanin et al., 2011). The His-rich motif of citrus CuCOR15 is able to bind Cu^2+^ (Hara et al., 2005), while VCaB45 in *Apium graveolens* binds Ca^2+^ in vacuoles (Heyen et al., 2002). Dehydrin ITP of *Ricinus communis* binds Fe^2+^ and Fe^3+^, allowing it to function as a metal ion transporter in plant phloem sap (Kruger et al., 2002). DHNs therefore also function as antioxidants by binding free metal ions and preventing excessive ROS formation in intracellular compounds since free metal ions act as catalyzers of ROS synthesis. HbDHN1 and HbDHN2 proteins contain 12 and 8 histidine residues, respectively; thus, whether these amino acid residues play a role in ROS scavenging requires further investigation.

As described above, the pleiotropic effects of dehydrins have been reported in response to various abiotic stresses, such as drought, high salinity, and cold. All three of the stresses cause dehydration and a reduction of the amount of free water available to the cell. However, the molecular mechanisms that underlie these effects remain unclear. As shown by Xie et al. the MtCAS31 dehydrin interacts with the ICE1 transcription factor to reduce stomatal density and enhance drought resistance (Xie et al., 2012), which suggests that DHNs can act as chaperones on other proteins under stresses. Classic chaperones not only prevent inappropriate protein aggregation but also form specific complexes with target proteins through interaction with hydrophobic patches. It is therefore relatively difficult for DHNs to establish specific interactions with other proteins especially under a dry state, explaining why some authors describe dehydrin protective functions based on non-specific protein-protein interactions (Tunnacliffe and Wise, 2007). Thus, as proposed by Graether and Boddington (2014), the gap between *in vitro* experiments and *in vivo* function has yet to be bridged.

## Conclusions

Induction of HbDHNs is an indicator of dehydration at the wounding site after mechanical wounding. Dehydration has been identified as a key signal of secondary laticifer differentiation in rubber trees. The data shown in this work suggest that HbDHN induction is not specific to the wounding response, but a common response to multiple abiotic stresses such as cold, salt, drought, wounding, ABA, ET, and JA treatment. Overexpression of HbDHNs increased tolerance to salt, drought, and osmotic stress in *Arabidopsis thaliana*, and compared with the WT, the transgenic plants accumulated more proline and suffered less membrane damage under salt and drought stress, and had a higher water content under osmotic stress. Moreover, the transgenic plants showed higher levels of antioxidant activity and accumulated less H_2_O_2_ and ${\text{O}}_{2}^{-}$. These findings suggest that HbDHNs act as a ROS scavenger, directly, or indirectly affecting laticifer differentiation. Both *HbDHNs* therefore have a pleiotropic effect on physiological processes, improving plant tolerance to multiple abiotic stresses.

## Author contributions

YC performed most of the experiments; XX performed transformation; MG and QY constructed the vectors and assisted the measurement of physiological index; XH supervised and wrote the manuscript. All authors have read and approved the final version of the manuscript.

### Conflict of interest statement

The authors declare that the research was conducted in the absence of any commercial or financial relationships that could be construed as a potential conflict of interest.

## Supplementary material

Table S1 **Primer sets used in this work**.

Table S2 **Comparison of cis elements in promoters of *HbDHN1* and *HbDHN2***.

Figure S1 **Phylogenetic tree of HbDHNs and dehydrins from other plant species**. The molecular phylogeny was constructed from complete protein sequence alignment of DHNs using the neighbor-joining method with bootstrap analysis (1,000 replicates). Bootstrap values are indicated.

## Abbreviations

ABA: abscisic acid
APX: ascorbate peroxidase
CAT: catalase
DAB: diaminobenzidine
DHN: dehydrin
DPI: diphenyleneiodonium chloride
ET: ethylene
JA: jasmonic acid
LEA: late embryogenesis abundant
NBT: nitro-blue tetrazolium
ROS: reactive oxygen species
SOD: superoxide dismutase.
