# Supplementary material for: Effect of HbDHN1 and HbDHN2 Genes on Abiotic Stress Responses in Arabidopsis
Source: Front Plant Sci. 2017 Apr 10;8:470. doi: 10.3389/fpls.2017.00470 (PMC5385384; doi:10.3389/fpls.2017.00470)
Supplement: Table S1 — Primer sets used in this work. [file Table1.DOC]

**Supplemental material**

**Table S1. Primer sets used in this work.**

| **Primer use** | **Primer sequence** |
| --- | --- |
| Full-length sequence | HbDHN1-F: 5’-ATGGCTGAGGAGCACAGCAAG-3’  HbDHN1-R: 5’-TTAATGGGACTCTTTCTCTT-3’;  HbDHN2-F: 5’-ATGGCGGAGCAGGATCACCA-3’  HbDHN2-R: 5’- CTATTCCTTCTTCTCTTCCT-3’ |
| Real-time  RT-PCR | HbDHN1RT-F:5’-TCGATCAGATAGCAGTTCTAGTTCTAG-3’ HbDHN1RT-R: 5’-CTTCTCCTTGATCTTCTCTTTCAATCC-3’;  HbDHN2RT-F: 5’-GAGGGAGTGGAGAAGAATAAGAAGAA-3’  *Hb*DHN2RT-R: 5’-CTCTTCCTTCTTTTCTTCCTTGTCTC-3’;  Actin2-F: 5’-TATGAATTACCCGATGGGCAAG-3’  Actin2-R: 5’- TGGAACAAGACTTCTGGGCAT-3’;  HbYLS8RT-F: 5’-CCTCGTCGTCATCCGATTC-3’  HbYLS8RT-R: 5’-CAGGCACCTCAGTGATGTC-3’;  Hb18SRT-F: 5’-GCTCGAAGACGATCAGATACC-3’  Hb18SRT-R: 5’-TTCAGCCTTGCGACCATAC-3’ |
| Plant expression | HbDHN1-F: 5’-CATGCCATGGCTGAGGAGCACAGCAAGA-3’  HbDHN1-R: 5’-AAGGTCACCTTAATGGGACTCTTTCTCTT-3’;  HbDHN2-F: 5’-CATGCCATGGCGGAGCAGGATCACCA-3’ HbDHN2-R: 5’-AAGGTCACCTATTCCTTCTTCTCTTCCT-3’ |
| Subcellular localization analysis | HbDHN1-F: 5’- GCGTCGACATGGCTGAGGAGCACAGCA-3’  HbDHN1-R: 5’-CGGGATCCATGGGACTCTTTCTCTTTC-3’;  HbDHN2-F: 5’-GCGTCGACATGGCGGAGCAGGATCACCA-3’ HbDHN2-R: 5’-cgGGATCCTTCCTTCTTCTCTTCCTCTG-3’ |
