# Supplementary material for: Effect of HbDHN1 and HbDHN2 Genes on Abiotic Stress Responses in Arabidopsis
Source: Front Plant Sci. 2017 Apr 10;8:470. doi: 10.3389/fpls.2017.00470 (PMC5385384; doi:10.3389/fpls.2017.00470)
Supplement: Table S2 — Comparison of cis elements in promoters of HbDHN1 and HbDHN2. [file Table2.DOC]

**Supplemental material**

**Table S2.** **Comparison of cis elements in promoters of *HbDHN1* and *HbDHN2*.**

| Cis-element | Sequence | HbDHN1 | HbDHN2 | Function |
| --- | --- | --- | --- | --- |
| ABRE | GCAACGTGTC | 4 | 1/1 | Abscisic acid responsiveness |
| ARE | TGGTTT | 1 | 2 | Cis-acting regulatory element essential for anaerobic induction |
| CGTCA-motif | CGTCA | 1 | 1 | Cis-acting regulatory element involved in MeJA-responsiveness |
| DRE/DRE-like | ACCGAC | 2/1 | 2 | Drought and cold responsiveness |
| ERE | ATTTCAAA | | 1 | Ethylene-responsive element |
| HSE | AAAAAATTTC | 1 | 1 | Heat stress responsiveness |
| LTR | CCGAC |  | 1 | Low-temperature responsiveness |
| MBS | TAACTG | 1 | 2 | MYB binding site involved in drought-inducibility |
| NACRS | CATGTG/CACG | | 1/3 | Drought, salt and ABA responsiveness |
| OCT | CGCGGATC | | 1 | Cis-acting regulatory element related to meristem specific activation |
| Skn-1_motif | GTCAT | 2 | 4 | Cis-acting regulatory element required for endosperm expression |
| TC-rich repeats | GTTTTCTTAC/  ATTTTCTTCA | | 3 | Cis-acting regulatory element involved in defense and stress responsiveness |
| TGACG-motif | TGACG | 1 | 1 | Cis-acting regulatory element involved in MeJA-responsiveness |
