# Supplementary figures and images for: Effect of HbDHN1 and HbDHN2 Genes on Abiotic Stress Responses in Arabidopsis
Source: Front Plant Sci. 2017 Apr 10;8:470. doi: 10.3389/fpls.2017.00470 (PMC5385384; doi:10.3389/fpls.2017.00470)

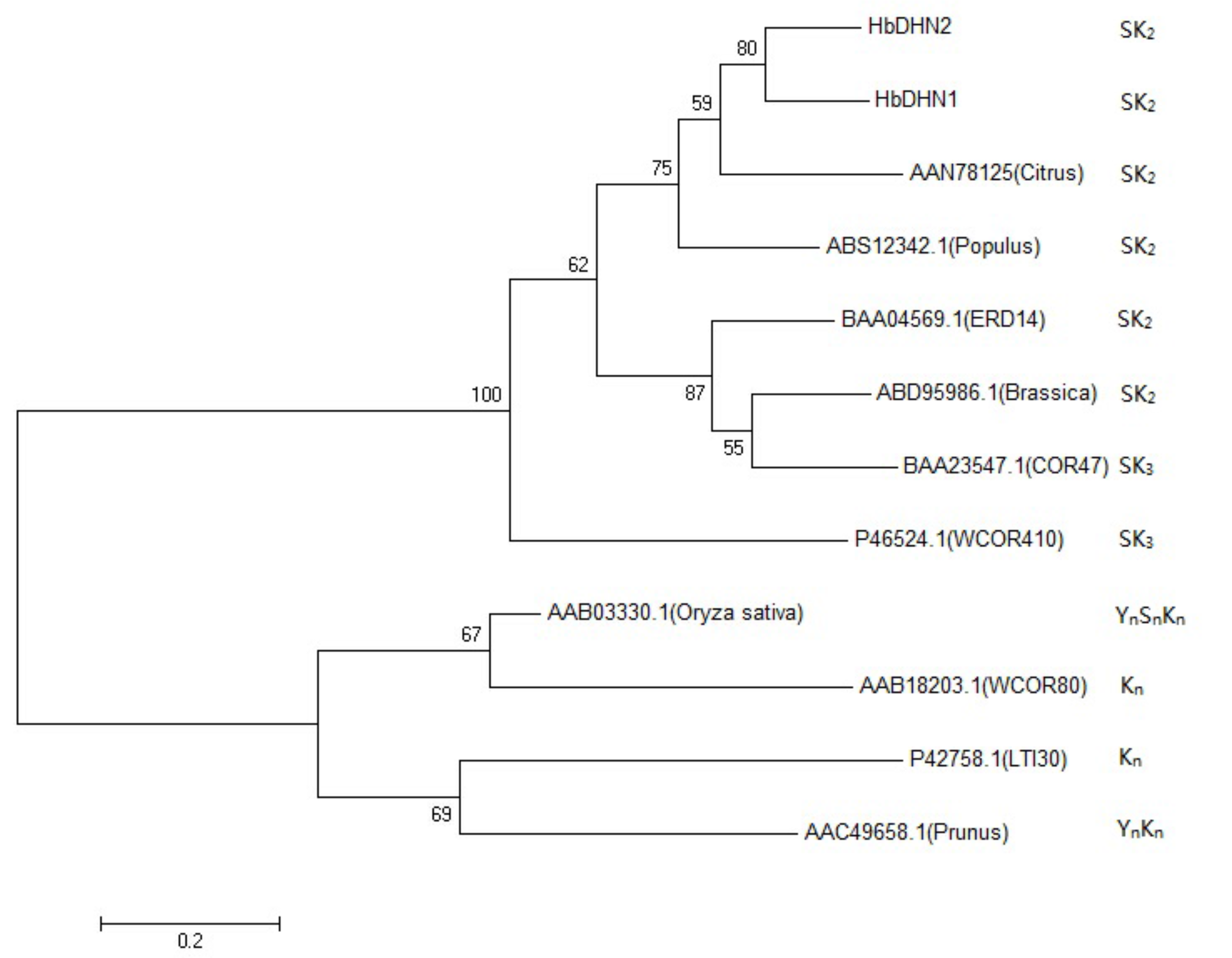

Supplement: Figure S1 — Phylogenetic tree of HbDHNs and dehydrins from other plant species. The molecular phylogeny was constructed from complete protein sequence alignment of DHNs using the neighbor-joining method with bootstrap analysis (1,000 replicates). Bootstrap values are indicated. [file Image1.TIF]
